# Supplementary material for: A Familiar Outbreak of Monophasic Salmonella serovar Typhimurium (ST34) Involving Three Dogs and Their Owner’s Children
Source: Pathogens. 2022 Dec 8;11(12):1500. doi: 10.3390/pathogens11121500 (PMC9788015; doi:10.3390/pathogens11121500)
Supplement: Supplementary file 1 [file pathogens-11-01500-s001.zip › pathogens-2042727-supplementary.pdf]

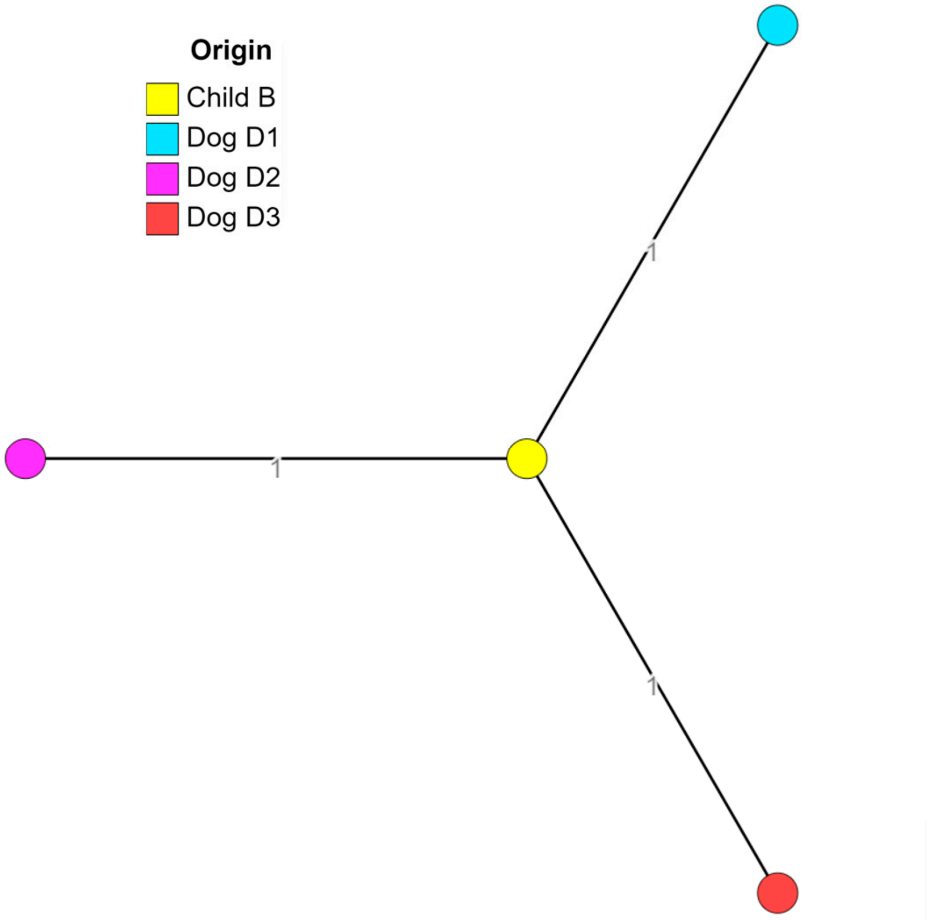

Figure S1. Minimum Spanning Tree (MST) of the cgMLST distances for the strains isolated in this work.

Table S1. Distance matrix of the cgMLST of the strains used in this work. The strains were isolated from the dogs (D1, D2, D3) and Child B (B).

**cgMLST**	**D1**	**D2**	**D3**	**B**
D1	0			
D2	2	0		
D3	2	2	0	
B	1	1	1	0
